# Diurnal Variation in P-glycoprotein-Mediated Transport and Cerebrospinal Fluid Turnover in the Brain

**DOI:** 10.1208/s12248-014-9625-4

**Published:** 2014-06-11

**Authors:** Laura Kervezee, Robin Hartman, Dirk-Jan van den Berg, Shinji Shimizu, Yumi Emoto-Yamamoto, Johanna H. Meijer, Elizabeth C. M. de Lange

**Affiliations:** 1 Division of Pharmacology, LACDR, Leiden University, PO Box 9502, 2300 RA Leiden, The Netherlands; 2 Laboratory for Neurophysiology, Department of Molecular Cell Biology, Leiden University Medical Center, PO Box 9600 Mailbox S5-P, 2300 RC Leiden, The Netherlands

**Keywords:** blood–brain barrier, chronobiology, intrabrain distribution, microdialysis, PBPK modeling

## Abstract

Nearly all bodily processes exhibit circadian rhythmicity. As a consequence, the pharmacokinetic and pharmacodynamic properties of a drug may also vary with time of day. The objective of this study was to investigate diurnal variation in processes that regulate drug concentrations in the brain, focusing on P-glycoprotein (P-gp). This efflux transporter limits the distribution of many drugs in the brain. To this end, the exposure to the P-gp substrate quinidine was determined in the plasma and brain tissue after intravenous administration in rats at six different time points over the 24-h period. Our results indicate that time of administration significantly affects the exposure to quinidine in the brain. Upon inhibition of P-gp, exposure to quinidine in brain tissue is constant over the 24-h period. To gain more insight into processes regulating brain concentrations, we used intracerebral microdialysis to determine the concentration of quinidine in brain extracellular fluid (ECF) and cerebrospinal fluid (CSF) after intravenous administration at two different time points. The data were analyzed by physiologically based pharmacokinetic modeling using NONMEM. The model shows that the variation is due to higher activity of P-gp-mediated transport from the deep brain compartment to the plasma compartment during the active period. Furthermore, the analysis reveals that CSF flux is higher in the resting period compared to the active period. In conclusion, we show that the exposure to a P-gp substrate in the brain depends on time of administration, thereby providing a new strategy for drug targeting to the brain.

**Electronic supplementary material:**

The online version of this article (doi:10.1208/s12248-014-9625-4) contains supplementary material, which is available to authorized users.

## INTRODUCTION

The treatment of brain tumors, infectious diseases, and various neurological disorders is often unsuccessful because the entry of most clinically available drugs into the brain is restricted (1). This is due to a number of protective mechanisms that prevent the distribution of potentially toxic compounds in the brain but, at the same time, impede drugs from reaching their target site in the central nervous system (CNS). For example, several types of efflux transporters are expressed in the brain, which expel a wide variety of exogenous substances from the brain back into the circulation (1,2).

A well-known efflux transporter in the CNS is P-glycoprotein (P-gp). At the level of the blood–brain barrier (BBB), it restricts the entry of many, mainly hydrophobic, drugs to the brain (3). Although P-gp is mostly known for its role in the efflux of its substrates at the BBB, it is also expressed in blood–cerebrospinal fluid (CSF) barrier (BCSFB) as well as in parenchymal cells types, such as astrocytes and pericytes (4). Several strategies have been proposed to diminish P-gp-mediated efflux, but it has been difficult to implement these strategies in the clinic and, especially, to apply them to chronic diseases (5).

The pharmacokinetic and pharmacodynamic properties of a wide variety of drugs show diurnal variation, which is a result of 24-h rhythms in, for example, gastrointestinal function, activity of xenobiotic-metabolizing enzymes, blood flow, and glomerular filtration rate (6). Consequently, therapeutic efficiency as well as the severity of side effects of drugs may vary with time of day (7–9). Regarding P-gp, it has been shown that its expression and activity show a diurnal rhythm in the intestines of rodents, leading to different plasma concentrations of orally administered P-gp substrates depending on the time of administration (10–15).

The objective of this study was to examine whether the distribution of P-gp substrates in the CNS depends on time of administration. Diurnal variation in P-gp activity could be exploited to either increase or reduce the effect of P-gp on CNS target site distribution, depending on whether the aim is to limit or enhance CNS distribution of the drug. Other processes that govern CNS target site concentrations (16), such as CSF turnover, may also vary over the course of the day. Understanding the effect of time of administration on CNS target site distribution may be useful to improve the efficiency of therapies involving P-gp substrates.

In this study, the concentration of the P-gp substrate quinidine was determined in plasma and brain tissue after intravenous administration at six different time points over the 24-h period in rats. As we observed a significant 24-h variation in P-gp-mediated drug transport in brain tissue, we next used intracerebral microdialysis to obtain quinidine concentration–time profiles in brain extracellular fluid (ECF) and in CSF at two different times of the day. Using physiologically based pharmacokinetic (PBPK) modeling to describe quinidine brain distribution (17), we were able to explore differences in P-gp-mediated transport and CSF flux between the active and resting period of the animals. The results show that time is an important consideration in the design of drug treatments targeted at the CNS.

## MATERIALS AND METHODS

### Animals

Male Wistar rats (Charles River, The Netherlands) were housed in groups under standard environmental conditions (12:12LD cycle) with free access to water and food (SDS, Technilab-BMI, Someren, the Netherlands). After surgery, the rats were kept individually. All animal procedures were performed in accordance with the Dutch law on animal experimentation and were approved by the Animal Ethics Committee of the Leiden University (protocol number DEC12088).

### Chemicals and Solutions

Quinidine, quinidine sulfate dehydrate, and quinine were obtained from Sigma-Aldrich (Zwijndrecht, the Netherlands); tariquidar from Xenova Group PLC (Cambridge, UK); saline and 5% glucose in saline from the Leiden University Medical Centre (Leiden, the Netherlands); Heparin from LEO Pharma BV (Breda, the Netherlands); nembutal and isoflurane from AUV (Cuijk, the Netherlands); phosphoric acid and boric acid from Merck (Darmstadt, Germany), sodium hydroxide and triethyl amine (TEA) from Baker (Enschede, the Netherlands); and methanol, methyl tert-butyl ether (TBME), and acetonitrile from Biosolve (Valkenswaard, the Netherlands). Perfusion fluid (PF) for microdialysis experiments was prepared as described in (18).

### Surgery

Animals were anesthetized by isoflurane during surgical procedures (induction, 5%; maintenance, 1–2%). A cannula was inserted in the femoral artery and femoral vein 7 days prior to the experiment as described previously (17). Animals used for microdialysis experiments also received microdialysis guides with dummy probes (CMA, Solna, Sweden) in the caudate putamen (CP; coordinates from bregma, AP-1.0 mm L + 3.0 mm V-3.4 mm) and in the cisterna magna (CM; coordinates from lambda, AP-2.51 mm L + 2.04 mm V-8.34 mm, at an angle of 25° anterior from the dorsoventral axis and 11° lateral from the anteroposterior axis) (17). Twenty-four hours before the experiment, the dummy probes in CP and CM were replaced by 4 and 1 mm microdialysis probes (CMA), respectively.

### Drug Administration, Serial Blood Sampling, and Collection of Brain Tissue

Brain distribution experiments were performed at six different time points across the 24-h period; *t* = 0 (start of quinidine administration) was at Zeitgeber Time (ZT) 0, ZT4, ZT8, ZT12, ZT16, or ZT20 (±10 min), with ZT12 defined as the moment of lights off. Microdialysis and *in vivo* recovery experiments were performed with *t* = 0 at ZT8 or ZT20. The number of animals per group is shown in Supplemental Table I. Between ZT12 and ZT0 experiments were conducted in dim red light.

Animals were pretreated with an intravenous infusion of vehicle (5% glucose in saline) or 15 mg/kg tariquidar in 5% glucose at *t* = −25 min for 10 min at a rate of 500 μL/min/kg with a syringe pump (Pump 22 Multiple Syringe Pump, Harvard Apparatus, Holliston, MA, USA). In brain distribution and microdialysis experiments, quinidine (10 mg/kg) was administered intravenously at *t* = 0 in 10 min at a rate of 250 μL/min/kg. Blood samples (100 μL) were collected at *t* = −10, 10, 30, 60, 90, 120, 150, 180, 210, and 240 min in heparinized Eppendorf tubes and centrifuged for 10 min at 5,000 rpm (Eppendorf Microcentrifuge Model 5415D) to obtain plasma. Plasma was stored at −20°C until analysis. At *t* = 240 min, the rats were sacrificed with an intravenous injection of Nembutal and transcardially perfused with PBS until the organs were free of blood. Brain tissue was removed and stored at −80°C.

### Intracerebral Microdialysis

Microdialysis probe inlets were connected to a syringe filled with PF, and the outlets were connected to a fraction collector (Univentor microsampler 820, Univentor Ltd, Zejtun, Malta) with FEP tubing. Perfusion of the microdialysis probes with a syringe pump (Bioanalytical Systems Inc., West Lafayette, USA) started 2 h prior to quinidine administration. Perfusion rate was set to 1 μL/min. Samples were collected every 20 min until *t* = 240 min and stored at −80°C.

### *In Vivo* Retrodialysis

The recovery of quinidine into the microdialysis probe was determined by *in vivo* retrodialysis. Probes were perfused with blank PF for 2 hours, after which the concentration of quinidine in PF was changed every 2 h from 20 to 50 ng/mL and to 200 ng/mL. Samples were collected every 20 min at a perfusion rate of 1 μL/min. The probe recovery (extraction fraction) was calculated as described by Westerhout *et al.* (2013). The extraction fractions from the probes located in CP and CM were used to convert the measured microdialysate concentrations to ECF and CSF concentrations, respectively.

### Plasma Protein Binding

To determine the degree of plasma protein binding of quinidine, 50 μL aliquots of each plasma sample taken at *t* = 30 min and *t* = 90 min were pooled per experimental time point (ZT) and pre-treatment (vehicle or TQD). The samples were equilibrated at 37°C for 30 min, and 200 μL was added to a pre-heated Centrifree 30 K ultrafiltration device (Millipore BV, Etten-Leur, the Netherlands). The samples were centrifuged for 20 min at 5,000 rpm at 37°C according to the manufacturer’s protocol. The unbound fraction of quinidine in plasma (f_unbound_) was calculated by dividing the concentration of the ultrafiltrate by the concentration in the unfiltered sample.

### Measurement of Quinidine Concentration in Microdialysate, Plasma, and Brain Homogenates

Quinidine concentrations in plasma, microdialysates, and brain homogenates were measured using high-performance liquid chromatography (HPLC) with fluorescence detection as described earlier (19). Microdialysates (10 μL) were directly injected into the HPLC system using a mobile phase with an acetonitrile/buffer ratio of 17:83 (*v*/*v*). Brain tissue was homogenized in 50-mM phosphate buffer (pH7.4) using the Bullet Blender 5 (Next Advance Inc., NY, USA) according to the manufacturer’s protocol. Brain homogenates were diluted 6× (*w*/*v*) in 50-mM phosphate buffer (pH7.4). Extraction of quinidine from plasma and brain homogenate was performed as described earlier (19). The injection volume was 2–20 μL depending on the type of sample. The mobile phase consisted of acetonitrile and buffer in a ratio of 14:86 (*v*/*v*).

### Data Analysis

Areas under the curve (AUC in ng/mL × min) of unbound quinidine in plasma from 0 to 240 min after administration (AUC_PLu,0–240_) were calculated using the trapezoidal rule. Ratios between parameters were determined for each animal individually before calculating the mean per group. The means of two groups were compared by an unpaired Student’s *t* test (normally distributed data) or a Mann–Whitney *U* test (not normally distributed data). To compare the means of more than two groups, an ANOVA (normally distributed data) or Kruskal–Wallis test (not normally distributed data) was used. Normal distribution was assessed by the Sharipo–Wilk test. *p* values below 0.05 were considered significant. Statistical analyses and graphical visualization were performed with R version 2.14.1 or version 3.0.1 (R Foundation for Statistical Computing, Vienna, Austria).

### Physiologically Based Pharmacokinetic Modeling

PBPK brain distribution modeling was performed to investigate the effect of time of administration on the pharmacokinetics of quinidine in plasma, total brain, ECF, and CSF quantitatively using a nonlinear mixed effect model approach with NONMEM software version VII (GloboMax LLC, Hanover, MD, USA). The PBPK model describing brain distribution of quinidine that was previously published (17) was applied to the data (Supplemental Figure 1). Data obtained during the microdialysis experiments in this study were added to the previously obtained data produced with the same experimental method during the resting period of the animals (17). Using covariate analysis, the effect of time of administration was tested on parameters describing P-gp-mediated transport and CSF flux. Statistical significance was based on changes in the objective function value (OFV; *p* < 0.05). The difference in the OFV obtained by comparing each model was assumed to be asymptotically chi-squared distributed with degrees of freedom (df) equal to the difference in the number of parameters between the two models. Goodness-of-fit plots (model predicted *vs.* observed data values) were evaluated by diagnostic scatter plots. The stability and performance of the final model were confirmed by a visual predictive check on the simulated distribution of the concentration data to cover over 90% of the observed data.

## RESULTS

### Time of Administration Has No Effect on Plasma Pharmacokinetics of Quinidine

**Fig. 1:**
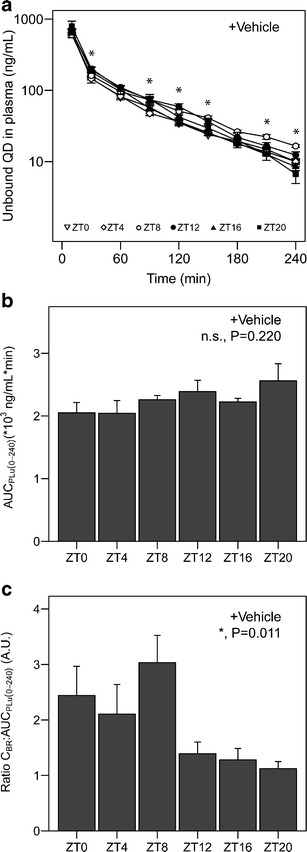
Quinidine exposure in plasma and brain in vehicle-treated animals. **a** Time–concentration profiles of unbound quinidine in plasma. *Different symbols* represent the different experimental time points (ZT0, 4, 8, 12, 16, and 20). *Asterisks* indicate significant effect of ZT on plasma concentration at the designated sampling times (*n* = 6–8 animals per group). **b** Area under the curve for unbound quinidine in plasma from 0 to 240 min after administration (AUC_PLu,0–240_) at different experimental time points (*n* = 5–8 animals per group). No significant time of day effect on AUC_PLu,0–240_ was found (*p* > 0.05, one-way ANOVA). **c** Quinidine concentration at *t* = 240 min in brain homogenate relative to plasma AUC (C_BR_:AUC_PLu,0–240_) at different experimental time points (*n* = 4–8 animals per group). Time of administration significantly affects this ratio (*p* < 0.05, Kruskal–Wallis rank sum test). Mean ± SEM in all graphs. *n.s.* not significant; *p < 0.05

We first determined the mean concentration–time profiles of unbound quinidine in plasma after intravenous administration at six experimental time points (ZT0-20) (Fig. 1a). Unbound plasma concentrations were derived from total plasma concentrations corrected for by the unbound fraction in plasma (0.286 ± 0.006). Total and unbound concentrations of quinidine in plasma showed a highly significant linear correlation (*r* ^2^ = 0.953, *N* = 24, *p* = 0.000, Pearson’s correlation; Supplemental Figure 2A). The unbound fraction did not depend on time of administration (F(5,17) = 2.52, *p* = 0.07, two-way ANOVA; Supplemental Figure 2B) and pre-treatment with TQD (F(1,17) = 1.70, *p* = 0.21, two-way ANOVA; Supplemental Figure 2C)

The unbound quinidine concentration in plasma in vehicle-treated animals at *t* = 30, 90, 120, 150, 210, and 240 was influenced by time of administration (*p* < 0.05, one-way ANOVA or Kruskal–Wallis, as appropriate; Fig. 1a). Time of administration had no significant effect on the area under the time concentration curve of unbound quinidine in plasma from 0 to 240 min after administration (AUC_PLu(0–240)_) (Fig. 1b F(5,34) = 1.49, *p* = 0.220, one-way ANOVA). These results indicate that the total exposure to quinidine in plasma is not affected by time of administration.

### Quinidine Brain Tissue Concentration Shows Significant Diurnal Variation

The concentration of quinidine in brain tissue at *t* = 240 (C_BR_) relative to AUC_PLu(0–240)_ was found to be significantly affected by time of administration (Fig. 1c H(5) = 14.9, *p* = 0.011, Kruskal–Wallis test). In experiments conducted in the resting period of the animals (*i.e.,* animals treated at ZT0, ZT4, and ZT8), the C_BR_: AUC_PLu(0–240)_ was on average twice as high compared to the ratio in the active period (at ZT12, ZT16, or ZT20). These results indicate that the exposure to the P-gp substrate quinidine in the brain, relative to plasma exposure, exhibits diurnal variation.

### Diurnal Variation of Total Brain Quinidine Concentrations is Due to the Variation in P-gp-Mediated Transport

**Fig. 2:**
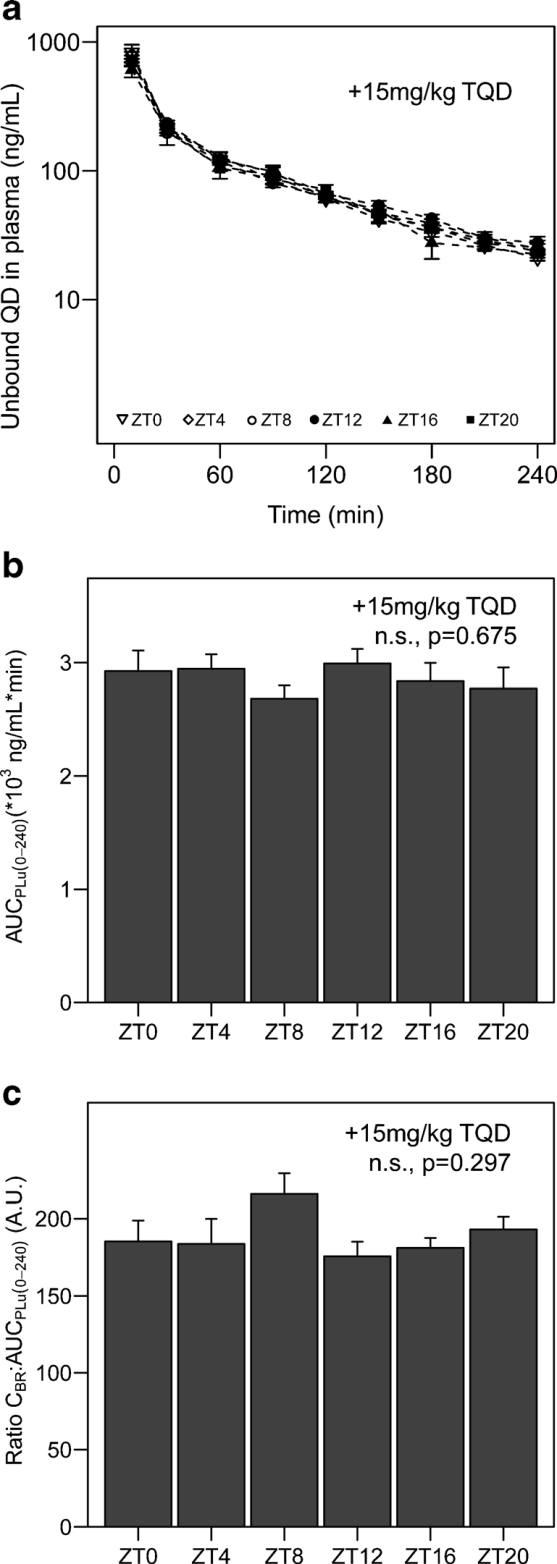
Quinidine exposure in plasma and brain in tariquidar-treated animals. **a** Time–concentration profiles of unbound quinidine in plasma. *Different symbols* represent the different experimental time points (ZT0, 4, 8, 12, 16, or 20). No significant effect of ZT was found between plasma concentrations at different sampling times (*n* = 6–8 animals per group). **b** Area under the curve for unbound quinidine in plasma from 0 to 240 min after administration (AUC_PLu,0–240_) at different experimental time points (*n* = 5–8 animals per group). No significant time of day effect on AUC_PLu,0–240_ was found (*p* > 0.05, one-way ANOVA). **c** Quinidine concentration at *t* = 240 min in brain homogenate relative to plasma AUC (C_BR_:AUC_PLu,0–240_) at different experimental time points (*n* = 4–8 animals per group). No significant time of day effect was found on this ratio in tariquidar-treated animals (*p* > 0.05, one-way ANOVA). Mean ± SEM in all graphs. *n.s.* not significant

To determine whether the effect of time of administration on the exposure to quinidine in brain tissue is due to variation in P-gp-mediated transport over the 24-h period, we administered the selective P-gp inhibitor tariquidar intravenously 30 min prior to quinidine administration at ZT0, 4, 8, 12, 16, or 20. Time of administration did not significantly affect unbound plasma concentrations of quinidine at any of the sampling points (Fig. 2a) and had no effect on AUC_PLu(0–240)_ (Fig. 2b F(5,33) = 0.635, *p* = 0.675, one-way ANOVA) in tariquidar-treated animals, indicating that also in this group of animals, time of administration does not affect exposure to quinidine in plasma. Importantly, time of administration also did not significantly affect C_BR_: AUC_PLu(0–240)_ of quinidine after tariquidar treatment (Fig. 2c F(5,31) = 1.28, *p* = 0.297, one-way ANOVA). Hence, tariquidar pre-treatment abolished the diurnal variation in C_BR_: AUC_PLu(0–240)_ that was observed in vehicle-treated animals. These results indicate that the exposure to quinidine in the brain relative to plasma levels exhibits diurnal variation unless P-gp is inhibited.

### Exposure to Quinidine in CNS is Influenced by Diurnal Variation in P-gp Activity and CSF Flux

Next, we used intracerebral microdialysis to determine unbound concentrations of quinidine in brain extracellular fluid (ECF) and cerebrospinal fluid (CSF) from 0 to 240 min after intravenous quinidine administration at ZT8 (resting period) and ZT20 (active period of the animals). The extraction fractions (4 mm probe, 13 ± 1.4%; 1 mm probe, 8.4 ± 2.6%) were used to calculate unbound concentrations in ECF and CSF from microdialysate concentrations.

**Fig. 3:**
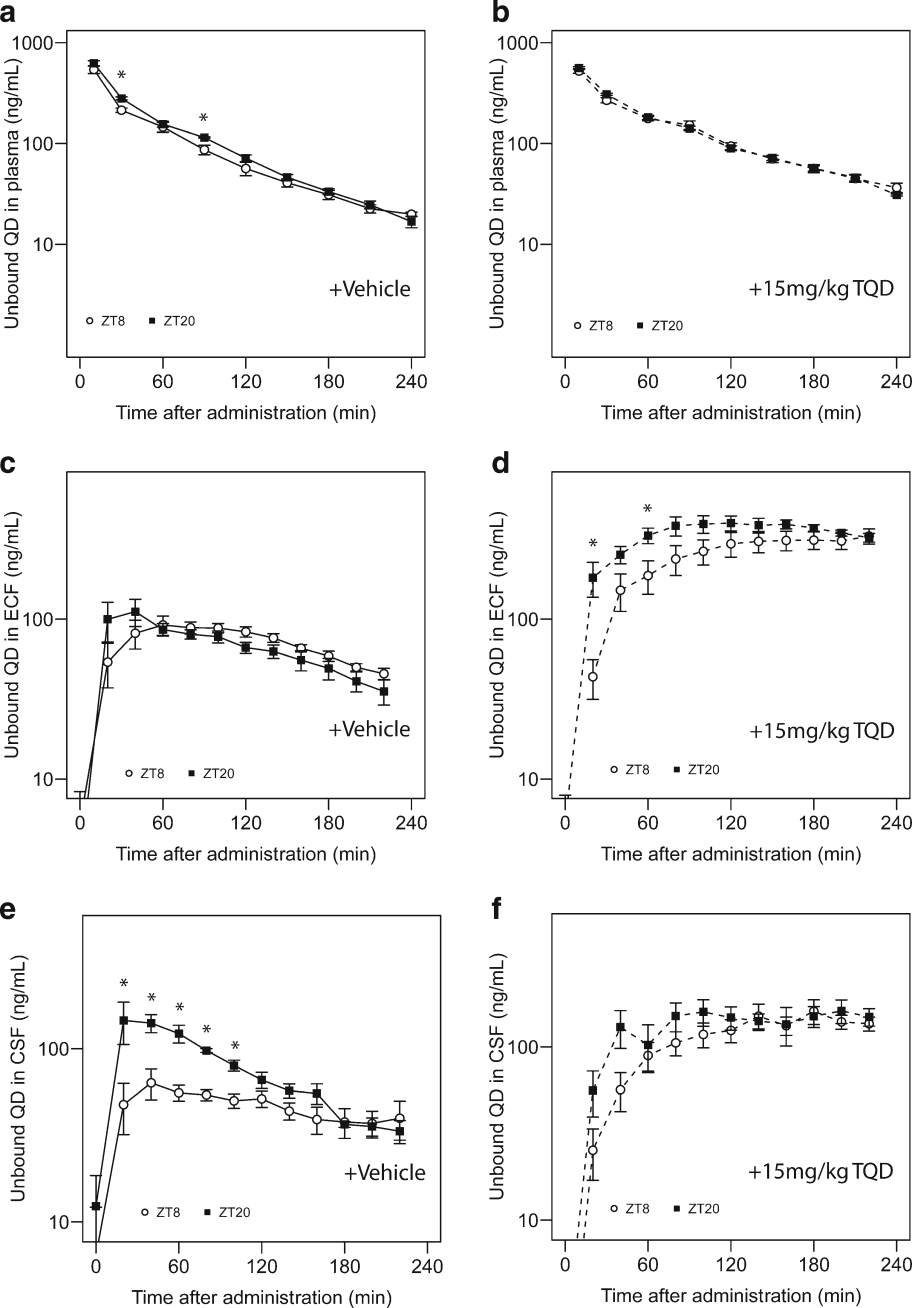
Microdialysis experiments in vehicle- and TQD-treated animals. Time–concentration profiles of unbound quinidine in plasma (**a**, **b**, *n* = 7–8 animals/group), ECF (**c**, **d**, *n* = 7–8 animals/group), and CSF (**e**, **f**, *n* = 6–8 animals/group) at ZT8 (*open symbols*) and ZT20 (*closed symbols*) in vehicle-treated animals (**a**, **c**, **e**) and TQD-treated animals (**b**, **d**, **f**). *Asterisks* indicate significant effect of ZT on quinidine concentration at the designated sampling times (*p* < 0.05, two-sided *t* test or Mann–Whitney *U* test). Mean ± SEM in all graphs. *p < 0.05

We calculated the mean concentration–time profiles of unbound quinidine in plasma, ECF, and CSF (Fig. 3) as well as C_BR_: AUC_PLu(0–240)_ (Supplemental Figure 3B-C) at ZT8 and ZT20 in vehicle- and TQD-treated animals. The fraction unbound of quinidine in plasma (0.276 ± 0.015) was not significantly different in these experiments compared to that observed in the brain distribution experiments (t(29) = 0.747, *p* = 0.461, two-sided *t* test, Supplemental Figure 2D). Also, AUC_PLu(0–240)_ and C_BR_: AUC_PLu(0–240)_ measured in these experiments were comparable to those obtained in brain distribution experiments (Supplemental Figure 3). In vehicle-treated animals, ECF concentrations were not significantly affected by time of administration at any of the sampling times (Fig. 3c), while CSF concentrations were higher in the first 100 min after administration in the active period compared to the values obtained after administration in the resting period (Fig. 3e). In tariquidar-treated animals, time of administration significantly affected ECF concentrations at two sampling times (Fig. 3d), but had no effect on CSF concentrations (Fig. 3f).

The data from the microdialysis experiments were fitted to a PBPK model that was previously used to describe quinidine pharmacokinetics in plasma, ECF, CSF, and deep brain tissue (17). We investigated the effect of time of administration on the parameters describing P-gp-mediated transport from the plasma compartment to and from deep brain tissue, ECF and CSF, and on CSF flux. Using covariate analysis, we found that the model that takes into account the effect of time of administration on both P-gp-mediated transport and CSF flux best described the data (Table I), indicating that time of administration significantly influences P-gp-mediated transport and CSF flux. The parameter estimates of the model (Table II) show that the estimated P-gp-mediated transport of quinidine from the deep brain compartment to plasma is almost 2.9× higher during the active period of the animals compared to the resting period (659 *vs* 228 μL/min, Table II). Furthermore, CSF flux is almost twice as high during the resting period of the animals compared to the active period (0.522 *vs* 0.227 μL/min, Table II).

**Table I:** Covariate Analysis

Covariate	Change in OFV^a^	*p* value
Time of administration (P-gp)	−155.6	<0.05
Time of administration (Q_CSF_)	−37.7	<0.05
Time of administration (P-gp and Q_CSF_)	−190.5	<0.05

^*a*^Change in objective function value (OFV) after addition of the covariate on various parameters compared to the model that does not take into account time of administration on any of the parameters

**Table II:** Parameter Estimates of the PBPK Model

Parameter			Value	RSE (%)
Estimates				
CL_E_	Passive		83.3 mL/min	10.3
	+P-gp		52.7 mL/min	6.5
Q_PL-PER1_	Passive		831 mL/min	9.6
Q_PL-PER2_	Passive		93.5 mL/min	18.3
CL_PL-Deep Brain_	Passive		982 μL/min	14.7
	+P-gp		NA^a^	
CL_Deep Brain-PL_	Passive		12.3 μL/min	19.8
	+P-gp	Resting period	228 μL/min	17.7
	+P-gp	Active period	659 μL/min	15.6
CL_PL-ECF_	Passive		25.7 μL/min	12.5
	+P-gp	Resting period	17 μL/min	18.5
	+P-gp	Active period	18.3 μL/min	18.5
CL_ECF-PL_	Passive		4.63 μL/min	15.2
	+P-gp	Resting period	3.14 μL/min	36.3
	+P-gp	Active period	3.98 μL/min	48.0
CL_PL-LV_	Passive		3.23 μL/min	19.9
	+P-gp	Resting period	1.55 μL/min	30.1
	+P-gp	Active period	2.44 μL/min	17.5
CL_LV-PL_	Passive		0.513 μL/min	24.0
	+P-gp		NA^a^	
CL_PL-CM_	Passive		0.753 μL/min	23.5
	+P-gp		NA^a^	
CL_CM-PL_	Passive		1.02 μL/min	33.7
	+P-gp		NA^a^	
Q_ECF_^b^			0.2 μL/min	
Q_CSF_		Resting period	0.522 μL/min	28.5
		Active period	0.227 μL/min	36.0
V_PL_^b^			10.6 mL	
V_PER_^1^			7.42 L	5.7
V_PER_^2^			7.09 L	17.3
V_Deep Brain_^b^			1440 μL	
V_ECF_^b^			290 μL	
V_LV_^b^			50 μL	
V_TFV_^b^			50 μL	
V_CM_^b^			17 μL	
V_SAS_^b^			180 μL	
Inter-individual variability				
CL_E_			33.2%	17.2
Residual error				
PL			42.8%	13.9
ECF			33.0%	13.9
LV			31.9%	18.7
CM			36.2%	13.8
Deep brain			35.6%	13.4
Objective function value			30,298.06	

*PER1* peripheral compartment 1, *PER2* peripheral compartment 2, *PL* plasma, *ECF* brain extracellular fluid, *LV* lateral ventricle, *CM* cisterna magna, *QECF* ECF flow, *QCSF* CSF flow, *V* volume of distribution of the compartments, *TFV* third and fourth ventricle, *SAS* subarachnoid space, *+P-gp* effect of P-gp-mediated transport on the parameter

^*a*^Parameter not available

^*b*^Physiological values derived from literature (17)

## DISCUSSION

In this study, we show that the exposure to the P-gp substrate quinidine in total brain tissue is subject to significant diurnal variation. This variation is abolished by pre-treatment with the selective P-gp inhibitor tariquidar, indicating a selective P-gp-mediated origin. The exposure to quinidine in brain tissue is lower during the active period compared to the resting period of the animals, which shows that the activity of P-gp is elevated during the active period. PBPK modeling based on plasma, ECF, CSF, and total brain concentrations of quinidine after intravenous administration in the active and resting period of the animals supports these findings by showing that time of administration significantly affects P-gp-mediated transport as well as CSF flux. Therefore, the findings presented in this study show that the exposure to a drug in the brain is affected by time of administration and provides a mechanism that involves 24-h variation in the activity of P-gp-mediated transport and CSF flux.

Circadian rhythms are present in many bodily processes and are controlled by a central clock located in the hypothalamus (20). Rhythms in peripheral organs are synchronized by the central clock, but are driven locally by an intracellular translational–transcriptional feedback loop with a period of approximately 24-h (21). This clock mechanism is responsible for the circadian rhythm that is found in the transcription, translation, and post-translational modification of many genes and their associated proteins (21). Our results show that the distribution of quinidine in the brain depends on time of administration due to variation in two processes. Firstly, we find that the estimated activity of P-gp-mediated transport is higher during the active period of the animals compared to the resting period. This is in line with several *in vitro* and *in vivo* studies that have shown 24-h variation in the expression and activity of P-gp in several different cell types (10–15). For example, Okyar *et al.* (2012) found that the activity of P-gp in the intestine is higher during the active period of rats (13). Future studies investigating the activity of P-gp in different cell types of the CNS across the 24-h cycle may provide further information on the molecular mechanisms underlying the findings presented in this study.

In addition to 24-h variation in P-gp activity, the PBPK model (17) that was used to describe the effect of time of administration on the pharmacokinetics of quinidine in the brain shows that CSF flow is larger during the resting period of the animals compared to the active period. In line with these findings, a recent study showed that sleep markedly increases CSF influx, thereby facilitating the removal of metabolic waste products from the CNS (22). This process may also affect the distribution of peripherally administered drugs in the brain. With the design used in this study, we cannot exclude the possibility that the sleep/wake state of the animal, rather than diurnal rhythmicity as such, is responsible for the variation in CSF flow or P-gp function over the 24-h period.

The variation in P-gp activity over the 24-h cycle had a much larger effect on quinidine concentrations in the brain tissue than in ECF. Total brain concentrations reflect the concentration of quinidine in both ECF and intracellular fluid (ICF) of brain parenchymal cells. The relatively small difference in the effect of P-gp on the clearance of quinidine from ECF to plasma during the active and resting periods of the animals cannot account for the large difference in the effect of P-gp on the clearance of quinidine from brain tissue to plasma. Therefore, we propose that an additional P-gp-dependent barrier between the ECF or plasma and the ICF of brain parenchymal cells gives rise to the observed variation. The existence of an additional barrier that affects the distribution of drugs in parenchymal cells has previously been suggested by the observation that valproic acid, an anticonvulsant drug, is subject to active efflux at the parenchymal cell membrane (23). Although valproic acid is not a P-gp substrate (24), Scism *et al.* (23) show that the parenchymal cell membrane can play an important role in the distribution of drugs in the brain. Furthermore, Syvänen *et al.* (19) show that induction of status epilepticus in rats affects P-gp activity at the level of the parenchyma rather than at the BBB.

Several lines of evidence suggest that P-gp is present in brain parenchymal cells, such as in pericytes and astrocytes (4,25,26). P-gp does not seem to be present in neurons in the healthy mammalian brain, but its expression is rapidly induced when challenged, for example, by hypoxic stress or the induction of status epilepticus (27,28). Importantly, it should be kept in mind that experiments are generally conducted during the resting period of the animals, which is the time window during which P-gp activity is lowest according to our findings. The existence of a barrier from the ECF to the ICF is of special interest for drugs that have an intracellular CNS target. Future studies should aim to elucidate this process more clearly.

A question that needs to be addressed is to what extent a twofold increase in total brain exposure is clinically relevant. Several strategies have been proposed to minimize the effect of P-gp on its substrates that are used as drugs to treat various CNS disorders. For example, pharmacological inhibition of P-gp by tariquidar in humans leads to a dose-dependent increase in brain uptake of a P-gp substrate (29,30). At a dose of 6 mg/kg, there is a fourfold increase in brain uptake of radiolabeled loperamide (29). However, this dose requires intravenous infusion for more than 1 h due to a hemolytic effect of one the co-solvents (29,31). Another limitation associated with P-gp inhibitors in humans is that the unbound plasma concentrations are relatively low, which limits the degree of P-gp inhibition (32). In light of these findings, the more than twofold increase in brain exposure that was observed in this study, by investigating a naturally occurring physiological process, could therefore well be of clinical relevance. This holds especially true for drugs with a narrow therapeutic index in which a slight increase in brain concentration leads to a large increase in effect size.

In summary, this study indicates that the exposure to a P-gp substrate in the brain is subject to diurnal variation. Using a selective and potent P-gp inhibitor, we are able to show that this is due to variation of P-gp-mediated transport which is markedly elevated during the active period of the animals compared to the resting period. Furthermore, CSF flux is increased during the resting period, which may also cause variations in the exposure to a drug in the brain. Importantly, these findings emphasize the need to take into account the timing of drug administration, both in clinical and experimental situations. Dosing at the appropriate time of the day may be an effective strategy to modulate the delivery of P-gp substrates to the brain.

## Electronic supplementary material

Below is the link to the electronic supplementary material.

Supplementary Table I (PDF 124 kb)

Supplemental Figure 1 (PDF 227 kb)

Supplemental Figure 2 (PDF 228 kb)

Supplemental Figure 3 (PDF 219 kb)
