# Supplementary material for: Diurnal Variation in P-glycoprotein-Mediated Transport and Cerebrospinal Fluid Turnover in the Brain
Source: AAPS J. 2014 Jun 11;16(5):1029–37. doi: 10.1208/s12248-014-9625-4 (PMC4147055; doi:10.1208/s12248-014-9625-4)
Supplement: Supplementary file 1 — (PDF 124 kb) [file 12248_2014_9625_MOESM1_ESM.pdf]

Supplemental Table I. Number of animals per treatment group

Experiment	ZT0	ZT4	ZT8	ZT12	ZT16	ZT20
Brain distribution						
Vehicle + 10mg/kg quinidine	7	8	5	8	8	5
15mg/kg tariquidar + 10mg/kg quinidine	7	7	6	7	6	5
Microdialysis						
Vehicle + 10mg/kg quinidine	ND	ND	8	ND	ND	6
15mg/kg tariquidar + 10mg/kg quinidine	ND	ND	8	ND	ND	7
In vivo retrodialysis						
Vehicle + 10mg/kg quinidine	ND	ND	1	ND	ND	2
15mg/kg tariquidar + 10mg.kg quinidine	ND	ND	1	ND	ND	1
