# Supplementary material for: Diurnal Variation in P-glycoprotein-Mediated Transport and Cerebrospinal Fluid Turnover in the Brain
Source: AAPS J. 2014 Jun 11;16(5):1029–37. doi: 10.1208/s12248-014-9625-4 (PMC4147055; doi:10.1208/s12248-014-9625-4)
Supplement: Supplementary file 2 — (PDF 227 kb) [file 12248_2014_9625_MOESM2_ESM.pdf]

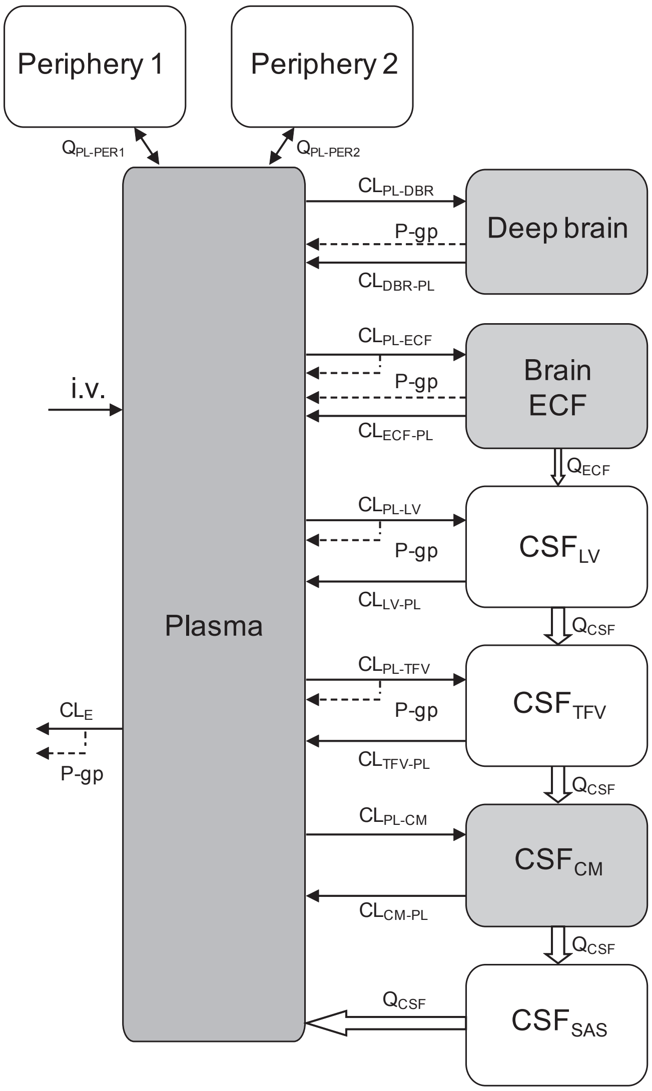

Supplemental Figure 1. Diagram of the PBPK model used to describe quinidine brain distribution. I.v. denotes the intravenous dose that enters the plasma compartment. CL_E is the elimination clearance from plasma. $Q_{PL-PER1}$ and $Q_{PL-PER2}$ are the clearances from the plasma compartment to the two peripheral compartments. Drug distribution from plasma to the brain is described by bi-directional clearance between the plasma compartment and the deep brain compartment (CL_{PL-DBR} and CL_{DBR-PL}), brain ECF compartment (CL_{PL-ECF} and CL_{ECF-PL}), and different CSF compartments (lateral ventricle: CL_{PL-LV} and CL_{LV-PL}; third and fourth ventricle: CL_{PL-TFV} and CL_{TFV-PL} and cisterna magna: CL_{PL-CM} and CL_{CM-PL}). Q_{ECF} denotes the flow rate of brain ECF to CSF and Q_{CSF} is the flow rate of CSF between the different CSF compartments (CSF_{SAS} refers to CSF in the subarachnoid space). Dashed arrows with the subscript P-gp represent P-glycoprotein mediated transport, which can either hinder drug influx or enhance drug efflux. The effect of P-gp on CL_{PL-CM}, CL_{CM-PL}, CL_{TFV-PL}, CL_{LV-PL} and CL_{PL-DBR} was not estimated, so these arrows were not included in the diagram. The grey physiological compartments indicate the data specifically obtained in the current study. All analyses were performed by using the subroutine ADVAN 6 and first-order conditional estimation with interaction.
