# Supplementary material for: Diurnal Variation in P-glycoprotein-Mediated Transport and Cerebrospinal Fluid Turnover in the Brain
Source: AAPS J. 2014 Jun 11;16(5):1029–37. doi: 10.1208/s12248-014-9625-4 (PMC4147055; doi:10.1208/s12248-014-9625-4)
Supplement: Supplementary file 3 — (PDF 228 kb) [file 12248_2014_9625_MOESM3_ESM.pdf]

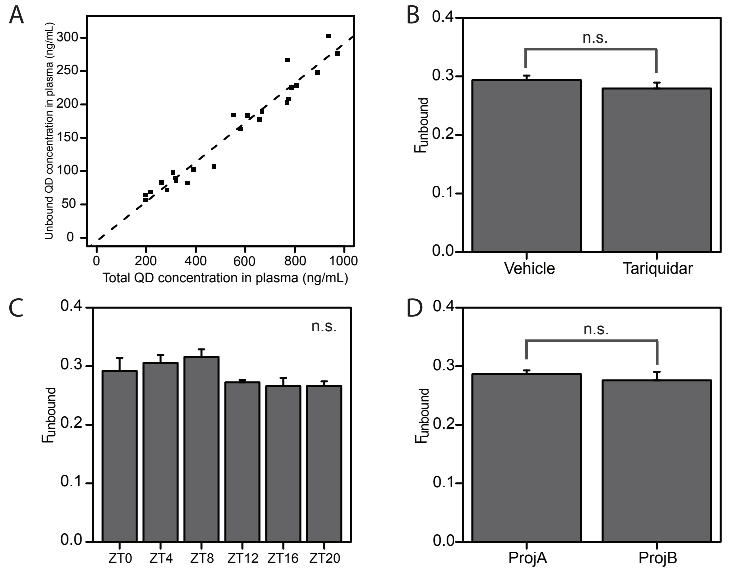

Supplemental Figure 2. Plasma protein binding. (A) Correlation between total and unbound quinidine concentrations in plasma from the brain distribution experiments. There is a strong linear correlation between total and unbound concentrations (r^2=0.953, N=24 samples, p=0.000). (B and C) Effect of tariquidar treatment (B, n=12 samples per group) and experimental time point (C, n=4 samples per group) on the unbound fraction of quinidine in plasma. A two-way ANOVA showed no significant effect of TQD treatment (TQD vs. vehicle: F(1,17)=1.70, p=0.21) and of time of administration (F(5,17)=2.52, p=0.07). (D) Effect of experimental set-up on the unbound fraction of quinidine in plasma. There is no significant difference in the unbound fraction between brain distribution (projA, n=24 samples) and microdialysis experiments (projB, n=7 samples) (t(29)=0.747, p=0.46, t-test). Mean±SEM in all graphs. N.s.: not significant.
