# Supplementary material for: Diurnal Variation in P-glycoprotein-Mediated Transport and Cerebrospinal Fluid Turnover in the Brain
Source: AAPS J. 2014 Jun 11;16(5):1029–37. doi: 10.1208/s12248-014-9625-4 (PMC4147055; doi:10.1208/s12248-014-9625-4)
Supplement: Supplementary file 4 — (PDF 219 kb) [file 12248_2014_9625_MOESM4_ESM.pdf]

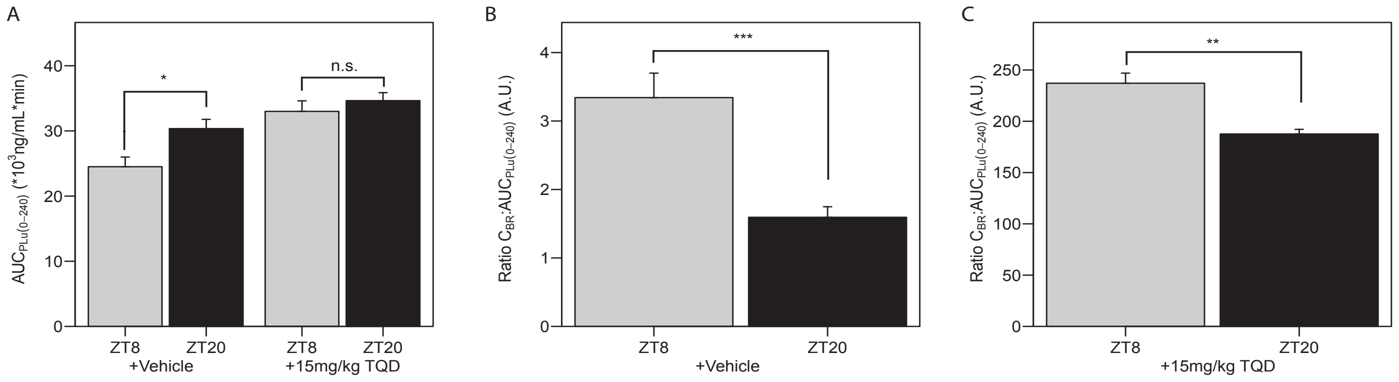

Supplemental Figure 3. Quinidine exposure in plasma and brain in the microdialysis group. (A) Area under the curve for unbound quinidine in plasma from 0 to 240 minutes after administration ($AUC_{PLu,0\text{-}240}$) at different experimental time points in vehicle and TQD treated animals (n=7-8 animals/ group). Time of administration had a significant on $AUC_{PLu,0\text{-}240}$ in vehicle treated animals (t(12)=-2.84, p=0.014, t-test), but not in TQD treated animals (t(13)=-0.79, p=0.44, t-test). (B-C) Quinidine concentration at t=240min in brain tissue relative to plasma AUC ($C_{BR:AUCPLu,0\text{-}240}$) at different experimental time points in vehicle (B) and TQD (C) treated animals (n=6-7 animals per group). Time of administration significantly affects this ratio in vehicle treated animals (t(11)=4.74, p=0.0006, t-test) and to a lesser extent in TQD treated animals (W=41, p=0.002). Mean±SEM in all graphs. N.s.: not significant; *p<0.05; **p<0.01; ***p<001.
